# Machine learning-based detection of workplace stress using wearable and multimodal data: a systematic literature review

**DOI:** 10.3389/frai.2026.1837195

**Published:** 2026-06-05

**Authors:** Luis Fernando Pareja Bernal, Souhir Ben Souissi, Christoph Golz

**Affiliations:** 1 Applied Machine Intelligence Research Group, School of Engineering and Computer Science, Bern University of Applied Science, Biel/Bern, Switzerland; 2 Applied Research and Development in Nursing, School of Health Professions, Bern University of Applied Science, Bern, Switzerland

**Keywords:** deep learning, machine learning, multimodal data, stress detection, wearable data, work related stress

## Abstract

Workplace stress is a significant concern, as it negatively impacts employee wellbeing and organizational productivity and is a major contributor to burnout and job turnover. Detecting stress in real-world work environments remains challenging; however, recent advances in machine learning and deep learning techniques offer promising solutions. Furthermore, the growing availability of multimodal data and wearable sensor technologies may facilitate individual stress tracking. In this paper, a systematic literature review is presented, focusing on machine learning and deep learning approaches for detecting workplace stress using wearable and multimodal data: (a) suggested machine learning and deep learning techniques are considered, (b) dataset characteristics and the sensor modalities employed are examined, (c) detection performance is reviewed, (d) workplace contexts and professional domains are discussed, and (e) gaps and future research directions are identified. The 20 selected studies show that machine learning and deep learning models applied to physiological, behavioral, and multimodal data can effectively detect workplace stress, particularly in high-risk occupations. However, limitations such as small sample sizes, limited dataset diversity, and minimal use of central nervous system signals (e.g., electroencephalogram) remain.

## 1 Introduction

Stress is a state of worry or mental tension arising from challenging situations (WHO, 2023). According to the World Health Organization, stress, depression, and anxiety result in approximately 12 billion lost workdays each year, with an economic impact of around US$1 trillion (World Health Organization, 2022). Additionally, stress can increase social challenges by straining interpersonal relationships, increasing susceptibility to substance abuse, and undermining community cohesion (Sinha, 2001; Randall and Bodenmann, 2009). Vulnerable populations often experience higher levels of stress due to factors such as socioeconomic disparities and limited access to resources (Turner and Avison, 2003).

Chronic stress also has serious health implications. It contributes to cardiovascular diseases, obesity, cancer, immune system suppression, and overall reductions in wellbeing (Steptoe and Kivimäki, 2013; Tomiyama, 2019). Mental health is similarly affected, with anxiety disorders and depression closely linked to prolonged stress exposure (Cohen et al., 2015). The economic burden associated with stress-related illnesses-including healthcare costs, lost productivity, and disability expenses-is substantial (Hassard et al., 2018).

In recent years, machine learning and deep learning techniques have been increasingly applied to detect stress in various contexts (Doki et al., 2021; Richer et al., 2024), leveraging physiological, behavioral, and multimodal data to identify stress patterns with promising accuracy. However, despite this growing body of research, comparatively little attention has been paid specifically to stress detection in **workplace environments**, where factors such as occupational demands, high-risk tasks, and organizational pressures create unique stress dynamics.

Several review articles have examined artificial intelligence (AI)-based stress detection, often focusing on **general** stress recognition across laboratory or non-specific environments (Yashaswini et al., 2025; Nandan et al., 2024; Lakshmanan et al., 2025; Vos et al., 2023; Taskasaplidis et al., 2024; Pinge et al., 2024). However, these reviews typically do not focus explicitly on workplace settings, nor do they systematically analyze key limitations related to occupational applicability and real-world deployment.

The goal of this systematic literature review is to examine the current state of the art in this research area, following the Preferred Reporting Items for Systematic Reviews and Meta-Analyses (PRISMA) (Page et al., 2021) guidelines. By systematically analyzing existing studies, this review aims not only to summarize the machine learning and deep learning approaches employed but also to highlight gaps in the literature, ultimately encouraging further research into multimodal and physiological machine learning solutions tailored to detecting stress in the workplace.

## 2 Related work

This section presents recent review articles on machine learning applications in stress detection at a general level. The selected works are briefly examined, followed by a synthesis of the machine learning techniques most commonly employed in stress-detection research. Finally, the relevance of our work in relation to the previously discussed reviews is outlined.

A number of review articles have been published on stress detection using machine learning over the last few years. A review published in 2025 (Yashaswini et al., 2025) focuses specifically on papers that use electroencephalography (EEG) data, which is recognized for its reliability, accuracy, and precision, and aims to build complete machine learning-based systems for forecasting stress levels. Another review, Nandan et al. (2024) also includes EEG-based studies but broadens the scope to innovative biomarkers such as salivary cortisol, Mineralocorticoid Receptor-Glucocorticoid Receptor (MR-GR) balance, and galvanic skin response (GSR). These features can all be measured from the head region, such as the head surface, forehead, and mouth. This review summarizes state-of-the-art approaches, identifies relevant biomarkers, and outlines how they relate to stress.

A broader perspective is presented in Lakshmanan et al. (2025), which examines not only physiological signals traditionally used for stress detection but also data-driven approaches involving EEG and electrocardiography (ECG). Additionally, it discusses stress indicators derived from wearable devices, including Galvanic Skin Response and Skin Temperature (ST). The paper further explores the use of machine learning and deep learning methods applied across multiple domains, including workplace settings, education, and automotive environments.

Another review, Vos et al. (2023) focuses on model generalization and evaluates how well-stress detection models perform when trained on publicly available datasets. Similarly, Taskasaplidis et al. (2024) provides a wide-ranging overview of stress detection methods involving the autonomic nervous system (ANS) and the hypothalamic-pituitary-adrenal (HPA) axis, including traditional biomarkers. This review goes further by considering how body movement and posture measurements may also relate to stress.

Finally, Pinge et al. (2024) examines the various types of sensors and wearable devices used to detect and monitor stress. It highlights the widespread reliance on classical physiological signals and discusses machine learning and deep learning techniques used to analyze them.

These related works have led to the identification of certain machine learning techniques often used in stress detection. A wide variety of techniques are namely employed, including support vector machine (SVM), linear regression, logistic regression, random forests, and more advanced techniques such as multilayer perceptron (MLP), long short-term memory (LSTM), convolutional neural networks (CNN), and eXtreme Gradient Boosting (XGBoost). This enumeration of machine learning techniques is not exhaustive, and only presents the techniques most frequently encountered when analyzing existing related works. It aims to provide the reader with an overview of machine learning techniques frequently used in stress detection.

While these previous reviews have examined stress detection in general contexts, covering laboratory settings, daily-life monitoring, multi-domain datasets, wearable devices, or EEG-based biomarkers, none have concentrated specifically on stress detection within real workplace environments. Existing reviews either focus on specific sensor modalities (e.g., EEG), broad physiological monitoring, or general affective computing, but they do not analyze how machine learning models are applied in the workplace, what types of workers are studied, which data modalities are collected in occupational settings, or how these models perform under real-world job conditions.

In contrast to the previously mentioned reviews, this systematic review addresses this gap by focusing on multimodal and physiological machine learning workplace stress detection and providing a structured examination of the current state of the art, identifying trends, limitations, and opportunities specific to occupational contexts.

## 3 Methods

This section describes the methodology used to conduct the systematic literature review. First, the employed methodology is outlined in Subsection 3.1, followed by the research questions guiding the review in Subsection 3.2. The selection of databases and the formulation of research queries, adjusted for different databases, are explained in Subsections 3.3 and 3.4. Next, the inclusion and exclusion criteria applied during the study selection process are detailed in Subsection 3.5. Finally, the overall selection process is presented with the help of a flow diagram in Subsection 3.6.

### 3.1 Methodology

The present systematic review was conducted following the (Page et al., 2021) guidelines. PRISMA is a widely accepted framework that provides tools such as checklists and flow diagrams to improve the transparency, rigor, and reproducibility of systematic reviews and meta-analyses. The main objective of applying this methodology is to enhance the quality and reliability of the review process. No registration number is associated with this review.

### 3.2 Research questions

The present systematic literature review is guided by the following research questions (RQs):

- **RQ1:** What machine learning and deep learning techniques are applied for workplace stress detection?
- **RQ2:** What types of data or sensor modalities are used in these studies?
- **RQ3:** What performance levels do these models achieve?
- **RQ4:** What workplace contexts are considered?

### 3.3 Databases

Papers published between 2017 and 2025 were considered for this review. This period was chosen to capture the most recent advances in workplace stress detection using machine learning (ML) and deep learning (DL), as significant developments in AI techniques and wearable/multimodal systems have occurred during these years.

- ACM Digital Library (ACM, 2025)
- IEEE Xplore (ieeexplore, 2025)
- Science Direct (Sciencedirect, 2025)
- Springer Link (Springer, 2025)
- PubMed (Pubmed, 2025)
- Google Scholar (Google, 2025)

These databases were chosen because they cover a broad range of computer science, engineering, and healthcare journals, ensuring comprehensive coverage of relevant literature.

### 3.4 Research query

To formulate the research query, the main concepts of workplace stress detection, machine learning, and wearable/multimodal data were first identified. Synonyms and related terms were then derived to ensure comprehensive coverage of the literature. Table 1 presents the identified concepts along with their respective synonyms and the final search query.

**Table 1:** Research concepts, synonyms, and search query used.

Concept	Synonyms/terms
Workplace stress	“Workplace stress,” “occupational stress,” “job stress,” and “work-related stress”
Stress detection	“Stress detection", “stress recognition”
Machine learning	“Machine learning", “deep learning”
Data modality	“Wearable,” “physiological,” “biometric,” “EDA,” “HRV,” and “multimodal”
Exclusion terms	“Review", “survey”

(The Query: “workplace stress” OR “occupational stress” OR “job stress” OR “work-related stress") AND (“stress detection” OR “stress recognition") AND (“machine learning” OR “deep learning") AND (“wearable” OR “physiological” OR “biometric” OR “EDA” OR “HRV” OR “multimodal") NOT (“review” OR “survey").

### 3.5 Eligibility criteria

The following criteria were applied to select studies for this systematic review. Table 2 summarizes the inclusion and exclusion criteria.

**Table 2:** Inclusion and exclusion criteria.

Inclusion criteria	Exclusion criteria
Studies published between 2017 and 2025	Studies published before 2017.
Studies focusing on workplace or occupational stress, defined as studies that explicitly state an intention to model, detect, or analyze stress in workplace or professional contexts.	Studies focusing on non-workplace stress (e.g., general population).
Use of machine learning or deep learning for stress detection.	Studies without ML/DL methods (e.g., only questionnaires, collection of data).
Studies using wearable or multi-modal data.	Studies using only self-reported stress without objective data.
Full-text articles in English.	Non-English publications, abstracts only, editorials, or reviews

For a paper to be included in this review, the terms defined in the research query had to appear in the title, keywords, or abstract. Duplicate records were excluded. The focus of this systematic review is on workplace stress detection using machine learning or deep learning, particularly in studies employing wearable or multimodal data. Studies that did not involve workplace settings or that relied solely on self-reported stress without objective measurements were excluded.

### 3.6 PRISMA flow diagram

**Figure 1:**
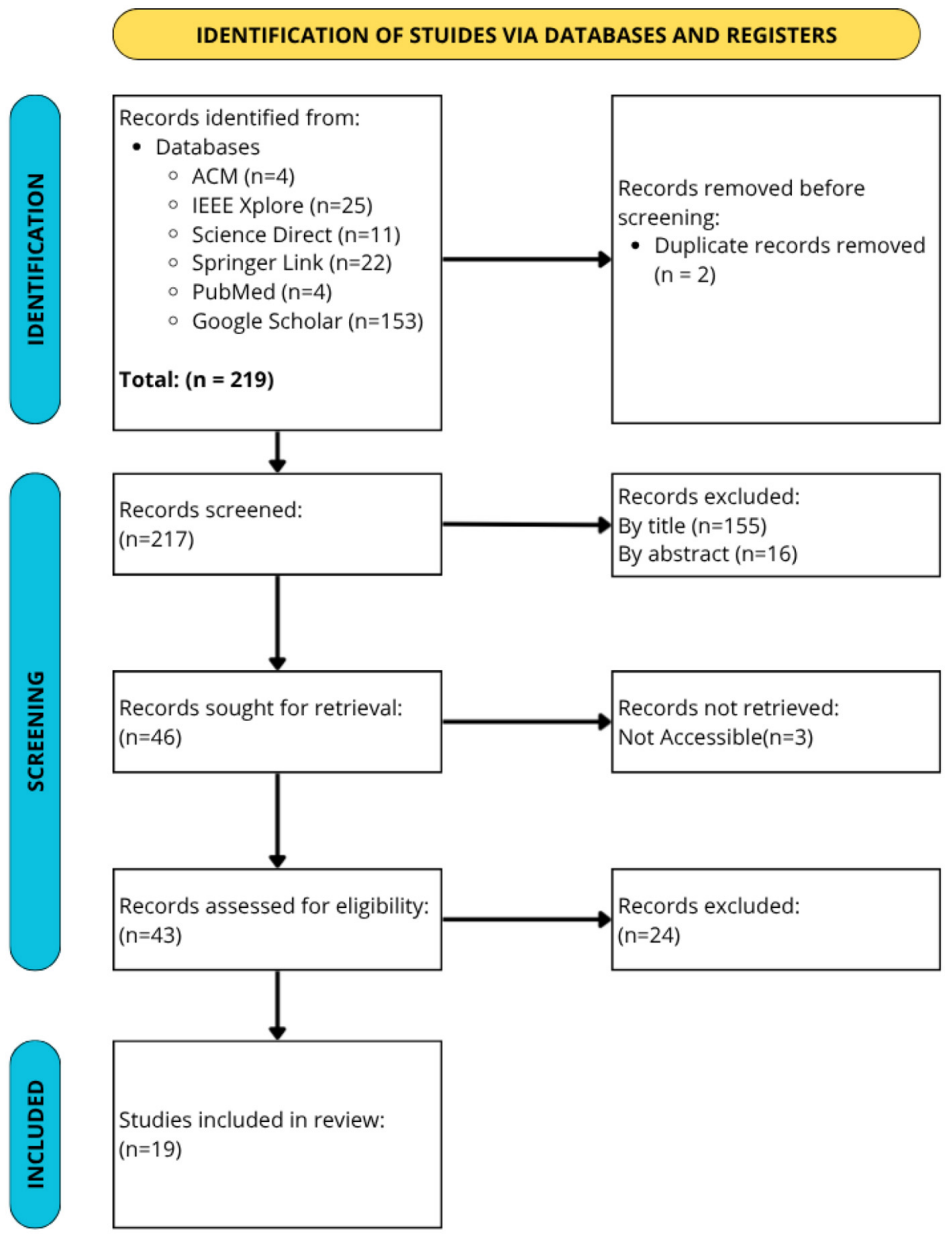
PRISMA flow diagram of the study selection process.

The study selection process is summarized in the PRISMA flow diagram (Figure 1). This diagram illustrates the number of records identified, screened, included, and excluded at each stage of the review.

The identification and screening processes were carried out between September and October 2025. During the identification phase, all papers resulting from the search queries across the selected databases were considered, totaling 219 records. The screening phase began with a manual analysis of paper titles, which led to the exclusion of 155 papers. Subsequently, abstracts were manually analyzed, resulting in the exclusion of 16 additional papers. After full-text reading of the remaining 43 papers, 24 were excluded because they did not meet the defined criteria, leaving 19 papers included in this systematic review.

The critical appraisal was conducted solely by the author, who worked independently in selecting studies. The most frequent reason for exclusion was that the papers did not focus on workplace stress.

## 4 Results

In this section, after presenting the distribution across different countries of the papers, we will analyze them according to the research questions defined in Subsection 3.2. Each research question is addressed in a separate subsection.

### 4.1 Country distribution

**Figure 2:**
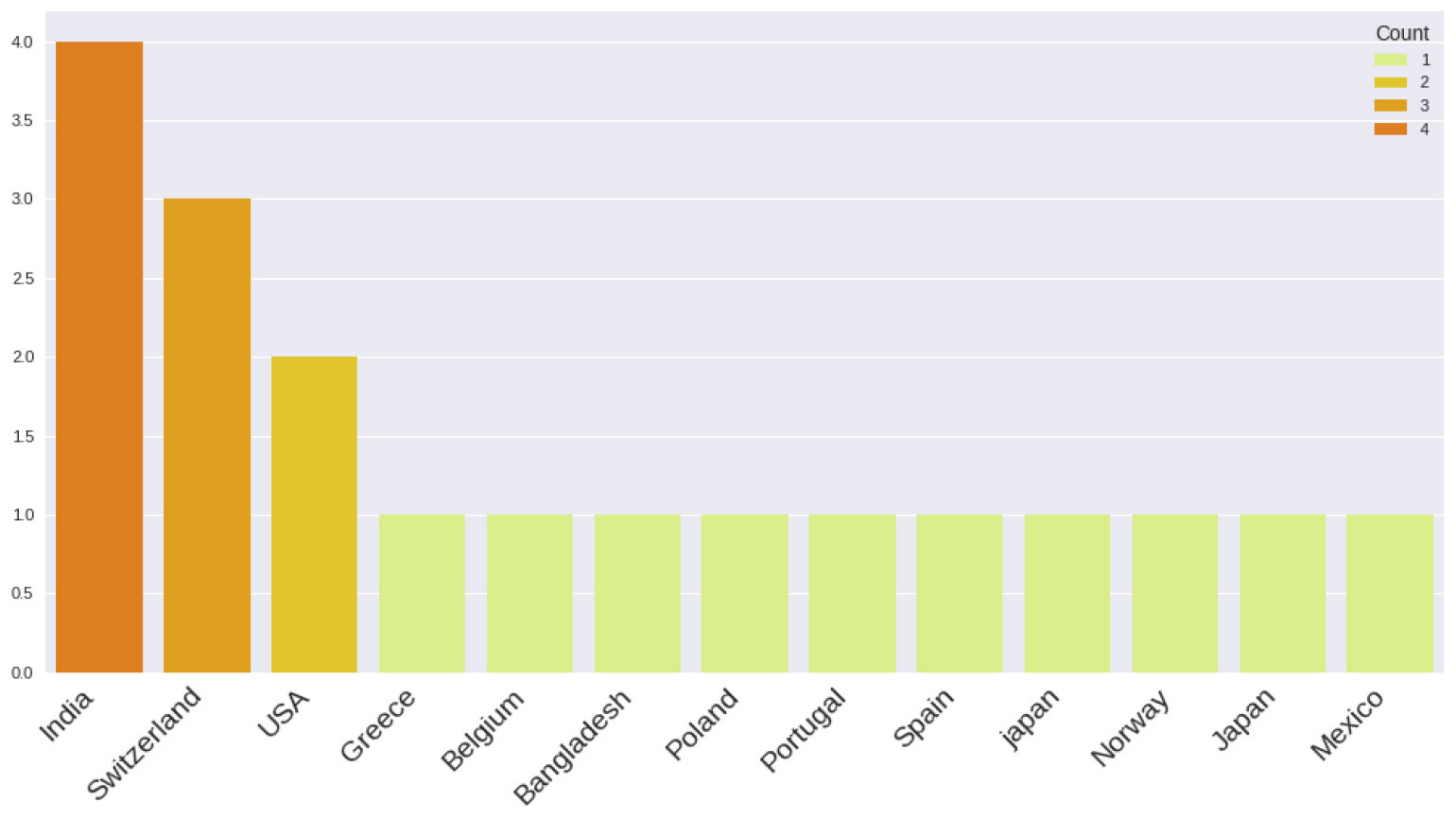
Country distribution of the included studies.

Figure 2 shows the distribution of the geographic locations of the authors. The country of the university or organization to which the author is affiliated is considered. If the authors of a paper have different geographic locations, the majority country is taken into consideration.

The geographical distribution of the analyzed studies shows that research on multimodal and physiological machine learning workplace stress detection is highly concentrated in a few regions. India is the most represented country, followed by several European nations such as Switzerland, Belgium, Poland, Portugal, Spain, and Norway, along with the United States, Japan, and Mexico.

This distribution reveals a noticeable lack of global diversity, as large parts of the world (particularly Africa, South America, and sections of Asia) are absent from the current research landscape. Such imbalance raises concerns regarding cultural and occupational bias, since stress responses and workplace dynamics differ significantly across countries. The dominance of studies from technologically advanced or research-intensive regions suggests that workplace stress detection remains an emerging focus primarily in countries with strong academic and innovation ecosystems.

Broader international participation would be essential for creating more generalizable and culturally robust stress detection models.

### 4.2 Research question 1: What machine learning and deep learning techniques are applied for work-place stress detection?

**Figure 3:**
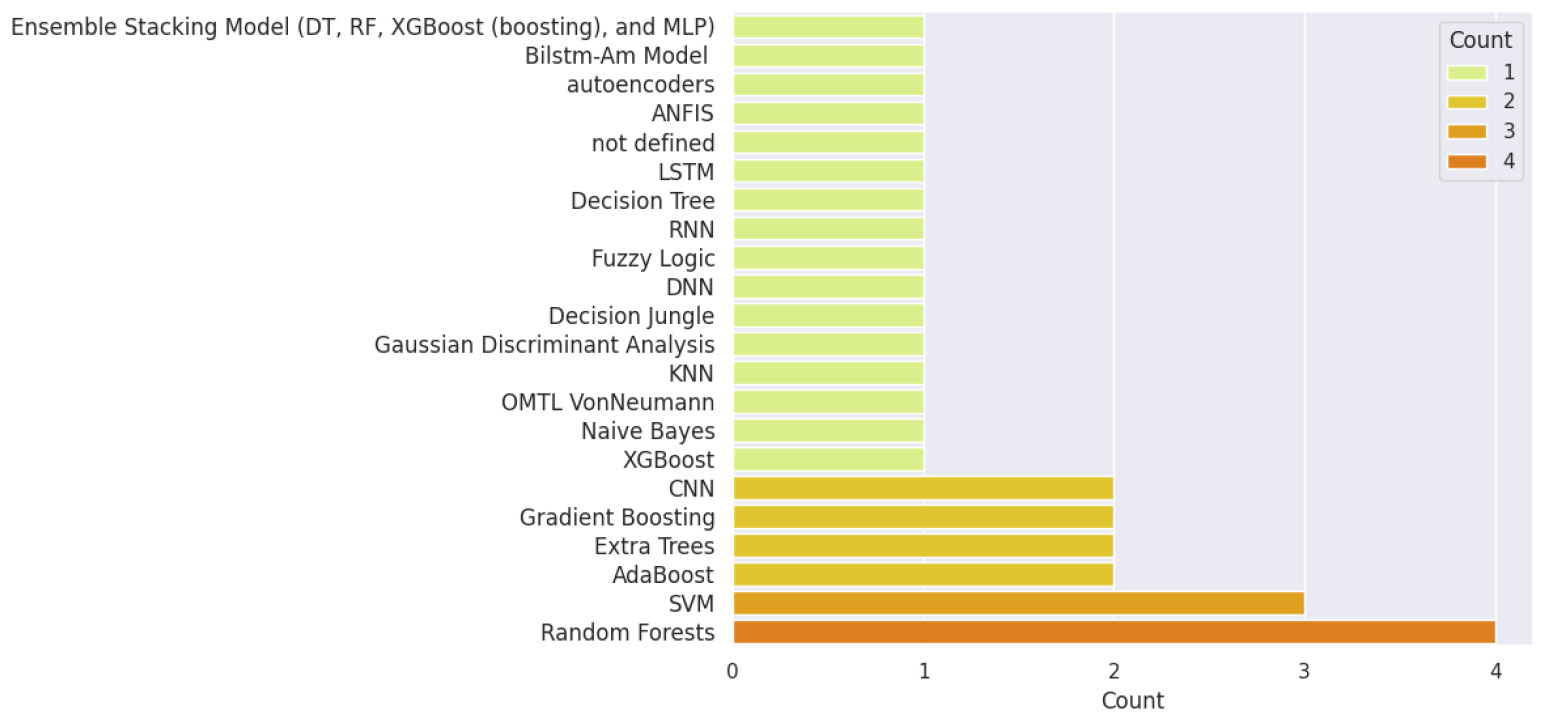
Distribution of machine learning models used in the reviewed studies.

The analysis of the selected papers revealed a diverse range of machine learning and deep learning techniques applied to workplace stress detection tasks. Figure 3 illustrates the distribution of the most frequently employed models across the included studies. Traditional machine learning approaches remain prevalent.

Among the most common techniques, Random Forests were the most frequently used model, appearing in four studies. CNNs and SVMs followed closely, each used in three studies. Other ensemble approaches, including Gradient Boosting, AdaBoost, and Extra Trees, appeared twice each, demonstrating the growing preference for boosting and bagging strategies to improve prediction accuracy and generalization.

Several papers also experimented with hybrid or more advanced deep learning methods. For instance, one study proposed an Ensemble Stacking Model combining Decision Trees, Random Forests, XGBoost, and Multi-Layer Perceptrons (MLP). Recurrent architectures such as bi-directional long short-term Memory and attention mechanism (BiLSTM-AM), recurrent neural network (RNN), and LSTM were also explored for modeling temporal dependencies in physiological signals.

Less frequent, yet notable methods included adaptive neuro fuzzy inference system (ANFIS) and Fuzzy Logic approaches, which leverage rule-based and adaptive systems for stress level classification. Other unique models included Deep Neural Networks (DNN), Autoencoders, and a Von Neumann-inspired Online Multi-Task Learning (OMTL) algorithm, each used in a single study. Classical classifiers such as Naïve Bayes, K-Nearest Neighbors (KNN), Gaussian Discriminant Analysis, Decision Tree, and Decision Jungle were also found in isolated works.

In general, the reviewed literature demonstrates that no single model dominates workplace stress detection research. Instead, the choice of technique often depends on the data modality, such as physiological signals or multi-modal inputs and the study's objective, whether focused on interpretability, accuracy, or real-time applicability.

### 4.3 Research question 2: What types of data or sensor modalities are used in these studies?

**Figure 4:**
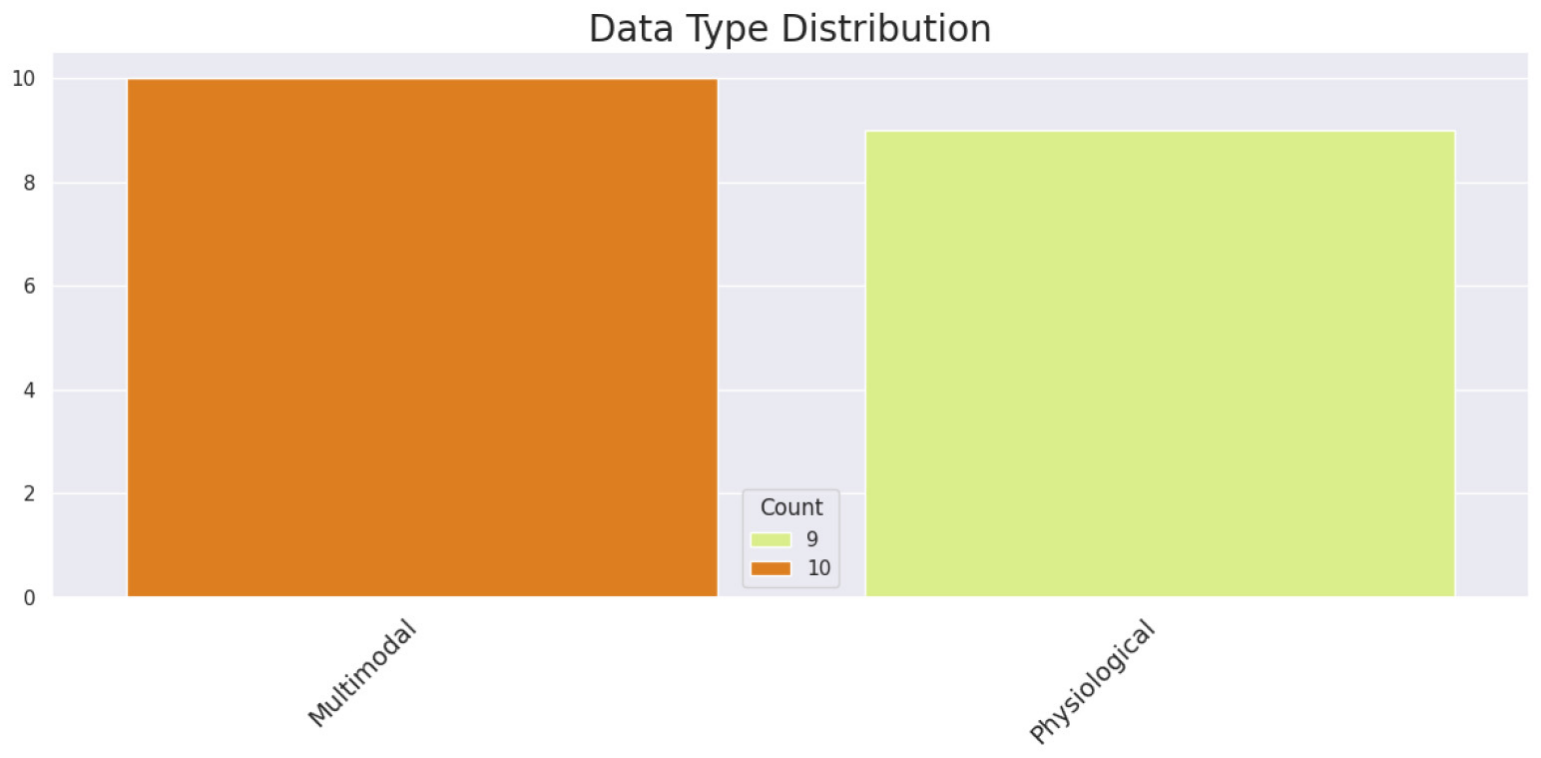
Distribution of data types used in the included studies.

Figure 4 shows the distribution of the types of data used across the studies.

Multimodal and physiological data were the most commonly used types, each appearing in nine studies. It is worth noting that most multimodal datasets also include physiological signals. This reflects a clear trend toward combining physiological measurements with other data sources to improve stress detection accuracy, while also highlighting the central role of physiological signals in this research area.

Physiological data was collected using a variety of sensor modalities, including:

- Heart Rate (HR)
- Heart Rate Variability (HRV)
- Electroencephalography (EEG)
- Blood Volume Pulse (BVP)
- Blood Pressure Levels (BPL)
- Body Temperature (BT)
- Electro-dermal Activity (EDA)
- Inter-beat Interval (IBI)
- Skin Temperature (ST)
- Skin Conductance (SC)
- Skin Conductance Response (SCR)
- Accelerometer (ACC)
- Motion Activity (MA)

These physiological signals allow researchers to capture subtle changes in the autonomic nervous system and other bodily responses that correlate with stress. Multimodal approaches often combine physiological signals with behavioral data, images, text, or other modalities to increase the reliability and robustness of stress detection.

#### Datasets

Table 3 summarizes the datasets used in the reviewed studies, with references provided where available. For each dataset, the dataset name and its availability, data modality, and corresponding signals and features are specified.

**Table 3:** Datasets used by the different papers.

Paper	Dataset source	Data modality	Details
Adamopoulos et al. (2025)	NOAA, NASA, and NCEI-NOAA (public datasets)	Multimodal	Surveys, real time environment data, sensor data (ambient temperature, humidity, pollutant levels), and historical workplace incident data
Mathur et al. (2024)	EmpathicSchool (Hosseini et al., 2022) (public dataset)	Physiological	EDA, HR, ST, ACC, IBI, and BVP
Pasha et al. (2024)	EmpathicSchool (Hosseini et al., 2022) (public dataset)	Physiological	EDA, HR, ST, ACC, IBI, and BVP
Rodrigues and Correia (2024)	Proprietary dataset	Multimodal	HRV, Questionnaire
Mihirette et al. (2025)	Affective road system dataset (Haouij et al., 2018), HCI lab dataset (Schneegass et al., 2013), WESAD (Schmidt et al., 2018), SWELL-KW (Koldijk et al., 2014), and Video rating dataset (public datasets)	Multimodal	EDA, HR, TEMP, BVP, ECG, EDA, HRV, SCR, and hand movement
Jebelli et al. (2019a)	Proprietary dataset	Physiological	EEG
Hossain et al. (2025)	Proprietary dataset	Physiological	EEG, HRV, and EDA
Jebelli et al. (2019b)	Proprietary dataset	Physiological	EEG
Jebelli et al. (2018)	Proprietary dataset	Physiological	EEG
Nkurikiyeyezu et al. (2019)	COMFORT and PHRENIC (Proprietary dataset) and SWELL-KW (Koldijk et al., 2014) (public dataset)	Multimodal	ECG, SC, HRV, computer logging, facial expressions, and body postures
Nägelin (2023)	Proprietary dataset	Multimodal	HRV and mouse and keyboard inputs
Martinez et al. (2018)	Proprietary dataset	Multimodal	Sleep data and questionnaire
Manikandan et al. (2024)	Not disclosed	Multimodal	Facial images and physiological signals
Oskooei et al. (2021)	Proprietary dataset	Physiological	HRV
Satapathy et al. (2022)	Proprietary dataset	Physiological	BPL and BT
Meelu and Assres (2024)	SWELL-KW (Koldijk et al., 2014) (public dataset)	Multimodal	ECG, SC, computer logging, and facial expressions
Vadym (2020)	Proprietary dataset	Physiological	EDA, HR, and MA
Bolliger et al. (2020)	Proprietary dataset	Multimodal	HR and smartphone sensor and usage data
Cvetković et al. (2017)	Proprietary dataset	Multimodal	BVP, HR, IBI, ST, EDA, and questionnaire

As observed in Table 3, physiological data dominates the literature, both as a single modality (Mathur et al., 2024; Pasha et al., 2024; Jebelli et al., 2019a; Hossain et al., 2025; Jebelli et al., 2019b, 2018; Oskooei et al., 2021; Satapathy et al., 2022; Vadym, 2020) and as a core component in multimodal approaches (Adamopoulos et al., 2025; Rodrigues and Correia, 2024; Mihirette et al., 2025; Nkurikiyeyezu et al., 2019; Nägelin, 2023; Martinez et al., 2018; Meelu and Assres, 2024; Bolliger et al., 2020; Cvetković et al., 2017). Many studies rely on a rich combination of physiological signals indicating a strong preference for capturing stress through internal biological responses rather than external or contextual factors.

Most studies created their own datasets (Rodrigues and Correia, 2024; Jebelli et al., 2019a; Hossain et al., 2025; Jebelli et al., 2019b, 2018; Nägelin, 2023; Martinez et al., 2018; Oskooei et al., 2021; Satapathy et al., 2022; Vadym, 2020; Bolliger et al., 2020; Cvetković et al., 2017), whereas some (Adamopoulos et al., 2025; Mathur et al., 2024; Pasha et al., 2024; Mihirette et al., 2025; Meelu and Assres, 2024; Schneegass et al., 2013; Schmidt et al., 2018) relied on publicly available datasets [Affective Road System Dataset (Haouij et al., 2018), SWELL-KW (Koldijk et al., 2014), EmpathicSchool (Hosseini et al., 2022), WESAD (Schmidt et al., 2018), Video Rating Dataset, data from National Oceanic and Atmospheric Administration (NOAA), National Aeronautics and Space Administration NASA and National Centers for Environmental Information (NCEI-NOAA)] and others used a combination of public and self-created datasets (Nkurikiyeyezu et al., 2019). All reviewed papers disclosed the dataset used for training except for paper (Manikandan et al., 2024).

### 4.4 Research question 3: What performance levels do these models achieve?

The reviewed studies report varying performance levels for workplace stress detection models. Table 4 summarizes the F1-scores and accuracies achieved by different machine learning and deep learning approaches.

**Table 4:** Performance of machine learning and deep learning models for workplace stress detection.

Model	Paper	F1-score (%)	Accuracy (%)
LSTM	Adamopoulos et al. (2025)	85.0	87.0
Decision tree	Mathur et al. (2024)	99.0	99.0
Bilstm-Am	Pasha et al. (2024)	95.0	96.0
Ensemble stacking model	Pasha et al. (2024)	98.0	97.0
AdaBoost	Rodrigues and Correia (2024)	77.5	77.8
Gradient boosting	Rodrigues and Correia (2024)	81.0	81.2
Random forests	Rodrigues and Correia (2024)	58.2	85.3
Extra trees	Rodrigues and Correia (2024)	84.1	84.4
Random forests	Mihirette et al. (2025)	62.0	–
DNN	Jebelli et al. (2019a)	–	86.6
Random forests	Hossain et al. (2025)	–	70.0
Extra trees	Hossain et al. (2025)	–	72.0
XGBoost	Hossain et al. (2025)	–	63.0
SVM	Hossain et al. (2025)	–	58.0
Naive bayes	Hossain et al. (2025)	–	80.0
OMTL VonNeumann	Jebelli et al. (2019b)	–	71.1
KNN	Jebelli et al. (2018)	–	65.8
Gaussian discriminant analysis	Jebelli et al. (2018)	–	74.9
SVM	Jebelli et al. (2018)	–	75.9
Decision jungle	Nkurikiyeyezu et al. (2019)	–	99.2
Gradient boosting	Nägelin (2023)	63.1	–
AdaBoost	Martinez et al. (2018)	98.5	–

“–” indicates that the metric was not reported in the original study.

Random Forests and Extra Trees are among the most frequently used classical machine learning models, often achieving high accuracy (between 70 and 85.3 %) but with mixed F1-scores (some of them not even reported). Gradient Boosting and AdaBoost also show strong performance, with F1-scores ranging from 63.1% to 81.0% and accuracy generally above 77%. SVM and KNN demonstrate moderate accuracy, though F1-scores are sometimes not reported. Decision Tree, despite being a classical model, achieved one of the highest performance levels in terms of both accuracy and F1-score, showing that interpretable models can still be very effective. Deep learning models like DNN, LSTM, and BiLSTM-AM achieve competitive results, with F1-scores as high as 95% and accuracies up to 96%–86%. Some ensemble approaches, like the Stacking Model combining decision trees, random forest, XGBoost, and MLP, show high performance, suggesting that combining multiple methods can enhance stress detection. More specialized architectures (e.g., OMTL VonNeumann, Decision Jungle) are less common but still achieve moderate to high accuracy.

#### 4.4.1 Observations

- Models from papers (Manikandan et al., 2024; Oskooei et al., 2021; Satapathy et al., 2022; Meelu and Assres, 2024), weren't include in the table because they didnt report any performance metric results
- Accuracy is the most frequently reported metric across studies, while F1-score is less consistently reported.
- Classical machine learning models tend to be more interpretable and still achieve high accuracy, whereas deep learning approaches offer slightly higher F1-scores, likely due to their ability to capture complex patterns in physiological or multimodal data.

Overall, both classical and deep learning models are effective for workplace stress detection. Table 5, Table 6 illustrate the top three models in terms of F1-score and accuracy, showing the variety of approaches available to achieve strong predictive performance in workplace stress detection.

**Table 5:** Top-performing models based on F1-score for workplace stress detection.

Model	Paper	F1-score (%)
Decision tree	Mathur et al. (2024)	99.0
Adaboost	Martinez et al. (2018)	98.5
Ensemble stacking model	Pasha et al. (2024)	98.0

**Table 6:** Top-performing models based on Accuracy for workplace stress detection.

Model	Paper	Accuracy (%)
Decision jungle	Nkurikiyeyezu et al. (2019)	99.2
Decision tree	Mathur et al. (2024)	99.0
Ensemble stacking model	Pasha et al. (2024)	97.0

### 4.5 Research question 4: What workplace contexts are considered?

The reviewed studies covered a variety of workplace contexts, though not uniformly. As shown in Table 7, several papers focused on specific occupational groups, including software engineers (IT sector), nurses and healthcare professionals (health sector), construction workers (construction sector), firefighters and public health inspectors (public sector), and agriculturists (agriculture sector). These studies emphasize stress detection in professions characterized by high workload, safety-critical tasks, or exposure to unpredictable work conditions.

**Table 7:** Overview of the profession and sector of subjects across studies.

Number of papers	Profession of subjects	Sector
2 (Mathur et al., 2024; Hossain et al., 2025)	Nurses	Health
3 (Jebelli et al., 2019a,b, 2018)	Construction workers	Construction
1 (Nägelin, 2023)	Office employees	Undefined
1 (Adamopoulos et al., 2025)	Public health inspectors	Public
1 (Pasha et al., 2024)	Healthcare professionals	Health
1 (Oskooei et al., 2021)	Firefighters	Public
1 (Satapathy et al., 2022)	Farmers/agriculturists	Agriculture
1 (Vadym, 2020)	Public servants	Public
1 (Bolliger et al., 2020)	Academic staff	Education
1 (Manikandan et al., 2024)	Software engineers	Software/IT
2 (Meelu and Assres, 2024; Martinez et al., 2018)	Undefined	Undefined
4 (Cvetković et al., 2017; Rodrigues and Correia, 2024; Mihirette et al., 2025; Nkurikiyeyezu et al., 2019)	Generalized	Generalized

However, a number of studies (two in total) did not explicitly define a professional context, and this information could not be inferred from the datasets used.

Four additional studies used generalized datasets, that is, datasets that do not target a specific profession. This indicates that while some research targets occupationally unique stressors, many stress-detection models are still developed using context-neutral or undefined workplace settings.

Overall, workplace contexts vary widely, but most studies cluster around healthcare, construction and public service, with a considerable portion lacking explicit contextual definition. This highlights a gap in the literature: many stress-detection approaches are not yet validated in real-world, domain-specific workplace environments.

## 5 Discussion

In this paper, a systematic literature review following the PRISMA guidelines (Page et al., 2021) was conducted with the aim of identifying the current state of workplace stress detection using AI. A total of 19 papers were selected and analyzed according to the research questions defined in Subsection 3.2.

Unlike prior reviews that address general stress detection, this study highlights several critical gaps that are particularly relevant to real-world occupational settings.

First, the sectors that currently receive the most research attention, particularly high-risk occupations are discussed in Subsection 5.2. This is followed by an examination of the broad variety of other workplace contexts that remain underexplored, as presented in Subsection 5.3.

Subsection 5.4 addresses the lack of workplace-specific datasets, as many studies rely on general stress datasets not tailored to real-world occupational environments. The need for studies with larger and more diverse participant samples is discussed in Subsection 5.5. Finally, Subsection 5.6 highlights the potential for expanding the types of physiological and behavioral variables measured to improve model performance and ecological validity.

### 5.1 Comparative analysis of the reviewed studies

The reviewed studies differ mainly in the types of data they use, the models they apply, and the performance they report. Some studies rely on physiological signals such as heart rate, heart rate variability, and electrodermal activity. Others combine these with additional data sources such as EEG, behavioral data, or images. In general, studies that use multiple data modalities tend to achieve better results than those relying on a single type of data.

In terms of models, classical machine learning methods such as Decision Trees, Random Forests, boosting methods, and ensemble approaches are most commonly used. Only a smaller number of studies apply deep learning models such as CNNs, LSTMs, BiLSTM-AM, and DNN.

Reported performance varies significantly across studies. Some papers report very high results (up to 97%–99%), while most studies achieve more moderate performance, typically above 70%.

The majority of high-performing models in workplace stress detection are classical machine learning approaches, particularly tree-based ensembles.

Overall, direct comparison between studies is difficult because they use different datasets, methods, and evaluation setups. This limits the ability to identify a single best-performing approach and highlights the need for more standardized evaluation protocols in future research.

### Multimodal deep learning approaches for stress detection.

Within the studies included in this review, the use of multimodal deep learning approaches remains relatively limited. Only three papers (Adamopoulos et al., 2025; Jebelli et al., 2019a; Manikandan et al., 2024) employ deep learning techniques, specifically LSTM (Adamopoulos et al., 2025), DNNs (Jebelli et al., 2019a), and CNNs combined with RNNs (Manikandan et al., 2024).

Among these studies, one study (Jebelli et al., 2019a) employing a DNN uses physiological signals as input data, specifically EDA, HR, ST, ACC, IBI, and BVP. In contrast, Adamopoulos et al. (2025) does not rely on physiological signals; instead, it combines survey data, real-time environmental data (e.g., temperature, humidity, and pollutant levels), and historical workplace incident data. For Manikandan et al. (2024), limited details are provided regarding the input data, although a CNN- and RNN-based approach is reported.

The reported performance achieved by these models was promising. The LSTM model trained on surveys and real-time environmental data achieved an accuracy of 87%, while the DNN model trained on physiological signal data achieved an accuracy of 86.6%. Although these results are not the highest reported among the reviewed models, they nevertheless demonstrate promising potential for stress detection in workplace settings using multimodal deep learning approaches.

### 5.2 Importance of high risk occupations in stress detection

As shown in Subsection Table 7, when excluding studies with undefined professions or generalized populations, approximately 53% of the included papers focus on high-risk workers (Hossain et al., 2025; Jebelli et al., 2018, 2019b; Pasha et al., 2024; Oskooei et al., 2021; Mathur et al., 2024; Jebelli et al., 2019a). High-risk professions tend to show stronger and more consistent physiological stress responses, which results in clearer and more distinguishable stress patterns. While these pronounced patterns don't directly translate to low-risk occupations, they provide valuable insights that can guide feature selection, sensor choices, and modeling strategies. Consequently, knowledge derived from high-risk workers can support the development of models capable of detecting the more subtle and heterogeneous stress responses typically observed in low-risk workplace environments.

### 5.3 Room for other workplace contexts and professions

From the 19 papers included in this review, only nine distinct professions were represented. Additionally, during the PRISMA filtering process, a large number of papers had to be excluded because their data did not originate from workplace environments. This highlights a substantial gap: many workplace contexts remain unexplored despite their potential to contribute valuable insights into stress detection patterns.

Even within sectors that do appear in the literature, the representation is narrow. For example, while several studies include nurses, other closely related professions such as doctors, surgeons, paramedics, or laboratory technicians are entirely absent. Similar gaps exist in other sectors, where only one role is typically studied despite the presence of diverse occupations that may experience stress differently.

This limited coverage suggests that current research captures only a small fraction of real-world workplace variability. Expanding the range of professions studied would not only improve generalizability but also enable the development of more robust and occupation-specific stress detection models.

### 5.4 Lack of suitable datasets

A noticeable pattern in some of the analyzed studies is the usage of pre-existing stress detection datasets such as the SWELL dataset (Koldijk et al., 2014) and WESAD (Schmidt et al., 2018). Several papers in our review (Mihirette et al., 2025; Meelu and Assres, 2024; Nkurikiyeyezu et al., 2019) make use of these datasets, despite their limitations in terms of workplace relevance and diversity.

The SWELL dataset, while valuable, suffers from limited variability. It includes only office workers, involves approximately 3 h of recording per participant, and contains data from just 25 individuals. This narrow scope restricts its applicability to broader workplace contexts and reduces the generalizability of models trained on it.

Similarly, the WESAD dataset was designed to detect general affective states in a laboratory setting rather than workplace stress. It contains data from only 15 participants and lacks any real-world professional context. Although WESAD appeared frequently among the papers excluded during the PRISMA screening, it was used in only one of the studies included in this review (Mihirette et al., 2025). This highlights a common issue in the field: many studies claim to investigate workplace stress detection but rely on datasets collected outside workplace environments, which undermines the ecological validity of their findings.

Overall, these limitations demonstrate a clear need for more suitable and robust datasets, ones that capture the variability of stress responses across different workplace environments, professions, and task demands. Without such datasets, the development of accurate and generalizable workplace stress detection models remains significantly constrained.

### 5.5 Need for more participants in the studies

As shown in Table 8, the majority of the reviewed studies rely on very small participant samples, with many experiments involving fewer than 30 individuals. Several studies—such as Hossain et al. (2025), Jebelli et al. (2019b), and Jebelli et al. (2018)—include only 7–20 participants, which is insufficient for capturing the variability of physiological or behavioral stress patterns across different workers. Not all papers where included in the Table 8, since not all of them mentioned explicitly how many participants they had in their data collection processes.

**Table 8:** Overview of the number of participants across studies.

Number of participants	Paper
25	Bolliger et al., 2020
15	Mathur et al., 2024
28	Rodrigues and Correia, 2024
200	Satapathy et al., 2022
8	Hossain et al., 2025
7	Jebelli et al., 2019b
7	Jebelli et al., 2018
20	Nkurikiyeyezu et al., 2019
18	Vadym, 2020
90	Nägelin, 2023
100 (Approx.)	Oskooei et al., 2021
26	Cvetković et al., 2017

Only three studies stand out for using larger samples: Satapathy et al. (2022) with 200 participants, Nägelin (2023) with approximately 100 participants, and Oskooei et al. (2021) with 90 participants. These represent the minority. Most other studies, including those that collected their own physiological measurements, remain limited in scale and therefore they could face challenges in model generalization.

Small participant samples introduce several problems. First, they reduce the generalizability of machine learning models. Stress responses are highly individual and influenced by factors such as age, physical condition, personality, and job role; with limited participants, it becomes difficult for models to learn patterns representative of the broader working population. Second, small datasets increase the risk of overfitting, where models perform well on the training data but fail to recognize stress effectively in real-world scenarios. Third, limited sample diversity restricts the ability to capture sector-specific stress patterns, which are particularly important for occupational stress detection.

Overall, these results demonstrate a clear need for future studies to involve larger and more diverse participant groups, ideally across multiple workplace sectors. Increasing sample sizes would improve model robustness, enhance cross-worker generalization, and ultimately support the development of AI systems that are reliable for real-world workplace stress monitoring.

### 5.6 Expanding measured variables

As seen in Table 3, most of the studies included in this review focus on peripheral physiological signals such as heart HR, HRV, and EDA. However, stress originates in the brain, particularly through activation of regions such as the amygdala, prefrontal cortex, and hypothalamus (Harvard Health Publishing, 2024), suggesting that central nervous system signals could provide more direct and informative indicators of stress.

One promising direction is the use of EEG, which provides real-time information on cognitive load, emotional arousal, and stress-related neural patterns (Acharya et al., 2025). Expanding the set of measured variables to include modalities like EEG offers several potential benefits. First, it can enable models to detect stress earlier, as neural markers precede physiological responses such as sweating or heart-rate changes. Second, EEG can help disambiguate stress from other states with similar peripheral signatures (e.g., physical activity), improving model specificity. Third, combining EEG with existing peripheral sensors could enable multimodal models that better capture the complexity of stress responses across diverse workplace environments.

Some studies have already explored this approach: (Jebelli et al., 2019a,b, 2018) used EEG-based monitoring to capture brain activity related to stress, demonstrating the feasibility and potential advantages of incorporating central nervous system measures in workplace stress detection.

While EEG-based measurement poses challenges, such as intrusiveness, cost, and limited feasibility for certain occupations, recent advances in dry-electrode, wearable, and low-profile EEG devices (Zhang et al., 2025; Xiong et al., 2025) make workplace integration increasingly realistic. Broadening the range of monitored variables, particularly toward brain-level indicators, may therefore be essential for developing more accurate, generalizable, and context-aware workplace stress-detection systems.

### 5.7 Key challenges in workplace stress detection

Based on the findings of this review, several key challenges in machine learning-based detection of workplace stress using wearable and multimodal data can be identified.

First, the current literature focuses mostly on a limited set of professions, particularly high-risk occupations, leaving many workplace contexts underexplored.

Second, the lack of workplace-specific datasets and the reliance on laboratory or general stress datasets limit the ecological validity of existing models.

Third, many studies are based on small participant samples, which restricts generalizability and increases the risk of overfitting.

Finally, the range of measured variables remains limited, with relatively few studies exploring central nervous system approaches (e.g., EEG). Addressing these challenges is essential for developing robust and scalable stress detection systems suitable for real-world workplace environments.

## 6 Conclusion

### 6.1 Key findings

This systematic literature review identified several key findings on the current state of artificial intelligence for workplace stress detection. Across the studies, both classical machine learning methods (such as Random Forests, SVM, Gradient Boosting, and Decision Trees) and deep learning techniques (including CNNs, LSTMs, autoencoders, and DNNs) were widely adopted. Many models achieved promising performance levels, with several reporting accuracies above 90%, demonstrating the feasibility of AI-driven stress detection in controlled and semi-controlled environments. The analysis of data types and sensor modalities revealed that most studies relied on physiological signals such as HR, HRV, EDA, BVP, IBI, and temperature, often collected through wearable sensors. Multimodal and physiological data represented the most common approaches. To enhance robustness, researchers frequently combined multiple physiological signals with behavioral indicators, environmental signals and questionnaire data. However, central nervous system data, particularly EEG, remains underutilized, despite evidence that stress originates in the brain (American Psychological Association, 2024). Only a few studies incorporated EEG, indicating an opportunity for expanding the range of measured variables to improve early detection and model specificity.

Regarding workplace contexts, the findings show a limited representation of professions. High-risk occupations such as healthcare workers, firefighters, construction workers, and public safety personnel appeared frequently, representing over half of the studies with clearly defined professions. While this focus is understandable due to the clearer physiological stress signatures in high-demand environments, it highlights the lack of research on low-risk or office-based occupations, which constitute a large portion of the workforce. Additionally, several studies relied on laboratory conditions or generic datasets such as SWELL and WESAD, which limits ecological validity and raises concerns about the generalizability of existing models to real workplace settings.

A recurring limitation across the reviewed studies is the small number of participants. Many experiments included fewer than 20 subjects, and even widely used datasets contained fewer than 30 participants. This restricts the statistical power, robustness, and ability of models to generalize across populations, job types, and cultural contexts. The geographic distribution of studies further reinforces this limitation, as most contributions originated from a small cluster of countries, leaving significant gaps in global representation.

### 6.2 Future directions

Overall, the findings underscore the promise of multimodal and physiological machine learning workplace stress detection but also highlight substantial gaps that must be addressed to advance the field. Future research should prioritize:

1. Larger and more diverse participant samples,
2. Workplace-specific datasets collected in real working environments,
3. Broader inclusion of professional sectors, including low-risk occupations,
4. Integration of central nervous system measurements, such as EEG, and
5. Cross-cultural and cross-context validation to ensure robustness.

By addressing these limitations, future systems can move closer to delivering reliable, real-time stress monitoring tools capable of supporting worker wellbeing, improving productivity, and enabling healthier workplace environments.

## Glossary

AdaBoost (Adaptive Boosting): A machine learning ensemble method that combines multiple weak learners–typically decision trees-by iteratively focusing on samples that previous learners misclassified.

Artificial Neural Network (ANN): A computational model inspired by biological neural networks, composed of layers of interconnected nodes that learn patterns through training.

Autoencoder: A type of neural network designed to learn efficient data representations by compressing input into a latent space and reconstructing it. Often used for anomaly detection or feature extraction.

BILSTM-AM (Bidirectional LSTM with Attention Mechanism): A deep learning architecture using bidirectional long short-term memory layers combined with attention to capture temporal dependencies and highlight important features.

Convolutional Neural Network (CNN): A deep learning model specialized for data with spatial structure (e.g., images, time series). Uses convolutional layers to detect local patterns.

Decision Jungle: A variation of decision trees where multiple nodes can share subtrees, forming a directed acyclic graph instead of a strict tree. Used for classification tasks.

Decision Tree (DT): A tree-structured model that makes decisions by splitting data based on feature thresholds. Simple, interpretable, and widely used.

Deep Neural Network (DNN): A neural network with multiple hidden layers, capable of learning complex hierarchical representations.

Electrodermal Activity (EDA): A physiological signal measuring changes in skin conductance related to sweat gland activity, widely used as a stress indicator.

Electroencephalography (EEG): A method for recording brain activity using scalp-mounted electrodes. Provides insight into cognitive and emotional states, including stress.

Ensemble Stacking Model: A meta-learning technique that combines multiple base models (e.g., Decision Tree, Random Forest, XGBoost, and MLP) and uses a final model to aggregate their predictions.

Extra Trees (Extremely Randomized Trees): An ensemble learning method similar to Random Forests but with more randomization in feature splits, often improving speed and robustness.

Fuzzy Logic: A method of reasoning that handles degrees of truth rather than binary values. Useful for modeling uncertainty in psychological states such as stress.

Gaussian Discriminant Analysis (GDA): A generative model that assumes each class follows a Gaussian distribution. Used for classification tasks.

Gradient Boosting Machine (GBM): An ensemble technique that builds models sequentially, where each new model corrects the errors of the previous ones.

Heart Rate Variability (HRV): A physiological measure reflecting variations in time between heartbeats. Highly correlated with stress and autonomic nervous system activity.

K-Nearest Neighbors (KNN): A simple machine learning classifier that predicts a label based on the majority class among the closest training samples.

Long Short-Term Memory (LSTM): A recurrent neural network capable of learning long-range temporal dependencies using memory cells that store information over time.

Multilayer Perceptron (MLP): An ANN composed of fully connected layers, often used as a baseline model for classification tasks.

Naive Bayes: A probabilistic classifier based on Bayes' Theorem with an assumption of feature independence.

OMTL— Online Multi-Task Learning (Von Neumann): A learning framework that simultaneously optimizes multiple related tasks over time. The Von Neumann divergence is used to guide regularization and updates.

Random Forest: An ensemble model built from multiple decision trees using bagging and feature randomness to improve performance and reduce overfitting.

Recurrent Neural Network (RNN): A type of neural network designed for sequential data, where outputs depend not only on the current input but also on previous states.

Support Vector Machine (SVM): A supervised machine learning model that finds the optimal separating hyperplane between classes, effective for high-dimensional data.

XGBoost (Extreme Gradient Boosting): A high-performance implementation of gradient boosting that uses regularization and parallelization, widely used in structured data tasks.
